# Ecology of Neglected Rodent-Borne American Orthohantaviruses

**DOI:** 10.3390/pathogens9050325

**Published:** 2020-04-26

**Authors:** Nathaniel Mull, Reilly Jackson, Tarja Sironen, Kristian M. Forbes

**Affiliations:** 1 Department of Biological Sciences, University of Arkansas, Fayetteville, AR 72701, USA; rtj006@uark.edu (R.J.); kmforbes@uark.edu (K.M.F.); 2 Department of Virology, University of Helsinki, 00290 Helsinki, Finland; Tarja.Sironen@helsinki.fi; 3 Department of Veterinary Biosciences, University of Helsinki, 00790 Helsinki, Finland

**Keywords:** *Hantaviridae*, hantavirus, HCPS, host-switch, reservoir, spillover, transmission

## Abstract

The number of documented American orthohantaviruses has increased significantly over recent decades, but most fundamental research has remained focused on just two of them: Andes virus (ANDV) and Sin Nombre virus (SNV). The majority of American orthohantaviruses are known to cause disease in humans, and most of these pathogenic strains were not described prior to human cases, indicating the importance of understanding all members of the virus clade. In this review, we summarize information on the ecology of under-studied rodent-borne American orthohantaviruses to form general conclusions and highlight important gaps in knowledge. Information regarding the presence and genetic diversity of many orthohantaviruses throughout the distributional range of their hosts is minimal and would significantly benefit from virus isolations to indicate a reservoir role. Additionally, few studies have investigated the mechanisms underlying transmission routes and factors affecting the environmental persistence of orthohantaviruses, limiting our understanding of factors driving prevalence fluctuations. As landscapes continue to change, host ranges and human exposure to orthohantaviruses likely will as well. Research on the ecology of neglected orthohantaviruses is necessary for understanding both current and future threats to human health.

## 1. Introduction

Due to their direct noticeable impacts on humans, certain viruses tend to receive relatively large amounts of research attention. Members of the *Coronaviridae* (SARS-CoV, MERS-CoV, and now SARS-CoV-2), *Filoviridae* (Ebola and Marburg virus), *Flaviviridae* (West Nile and Zika virus), *Lyssaviridae* (rabies), and *Paramyxoviridae* (Hendra and Nipah virus) families contain several dangerous human pathogens that have emerged in recent decades and have resulted in extensive research attention. While studying such viruses is important, there are an untold number of other pathogens that persist among humans and wildlife that receive little to no attention [1]. Even in high-profile viral groups, a disproportionate amount of attention is given to the viruses that are known to cause disease in humans, highlighted by the current global response to SARS-CoV-2. Due to unforeseeable circumstances, such as host-switching events (e.g., influenza virus, human immunodeficiency virus [2]), exposure to new viruses via landscape encroachment (e.g., Hendra virus [3], Nipah virus [4]), and changes in host or virus geographic range due to climate change, species introduction, or migration events (e.g., Zika virus [5], West Nile virus [6]), less-significant viruses can quickly become significant human health concerns. Therefore, viruses that are disproportionately under-studied require research focus, and they may ultimately aid understanding of related viruses and increase awareness of current and future threats.

A key example of research bias within a virus group is the hantavirus family (*Bunyavirales: Hantaviridae*). Recent taxonomic restructuring of hantaviruses was necessitated by the discovery of non-rodent- and non-mammal-borne viruses [7,8]. However, mammals, particularly rodents, are still the most common natural hosts of hantaviruses, encompassing viruses in the largest subfamily (*Mammantavirinae*) and genus *(Orthohantavirus*) [9], and only rodent-borne orthohantaviruses have been linked to human disease [10]. Human infections caused by spillover of Old World and New World orthohantaviruses can result in hemorrhagic fever with renal syndrome (HFRS) or hantavirus cardiopulmonary syndrome (HCPS or HPS), respectively [11]. 

The International Committee on Taxonomy of Viruses (ICTV) lists 58 unique orthohantaviruses distributed throughout the world, with 20 distinct viruses within 12 virus species endemic to North and South America [7,8,9]. Although the first known American orthohantavirus, Prospect Hill virus (PHV), was described in 1985 [12], most viruses were found shortly after the 1993 outbreak of Sin Nombre virus (SNV) [13] in North America and the 1995 cases of Andes virus (ANDV) [14] in South America (Table 1). New orthohantaviruses and genotypes continue to be identified via broad surveillance. Some discovered genotypes are suggested to be distinct viruses, but a lack of sequence data and virus isolation prevents formal taxonomic placement. For example, phylogenetic analyses show up to 10 distinct branches within the *Andes orthohantavirus* clade [15,16], but only four strains meet all ICTV criteria as distinct viruses (Table 1 and Table A1) [7,8,9].

Despite an increasing number of described hantaviruses, ANDV and SNV are disproportionately studied when compared to other orthohantaviruses in the Americas (Table 1). Such bias may be the reason for inadequate information to discriminate between potentially different viruses, and the lack of distinction may discourage the collection of additional data, creating a negative feedback loop. Muleshoe virus (MULV), for instance, is a genotype of Black Creek Canal virus (BCCV), and evidence supports MULV being a separate virus based on genetic differences [17]. However, the necessary ICTV criterion of MULV isolation has not been accomplished, which keeps MULV from being distinguished as a distinct virus strain and may limit the amount of research conducted on this genotype.

Until more virus-specific information is known, we must infer characteristics of under-studied orthohantaviruses using other available information. In this review, we summarize current knowledge on neglected orthohantaviruses and highlight areas where future research is necessary. To determine the potential range of these viruses, we report evidence regarding the rodent hosts of each American orthohantavirus and the potential for various host–virus relationships and communities based on existing evidence. Information regarding transmission for well-studied orthohantavirus systems is used to postulate the transmission characteristics of neglected American orthohantaviruses, including direct transmission routes, environmental persistence, and spillover risk to humans.

## 2. Host Diversity 

As the number of described orthohantaviruses increases, so does the number of suggested reservoir hosts (Table A1). Reservoir hosts typically have asymptomatic and persistent infections [11,19], although there is evidence of negative effects associated with orthohantavirus infection on the survival of young animals [20] and possibly decreased weight gain in newly-infected individuals [21]. Most studies that identify orthohantavirus infections in rodents have not evaluated the pathological or demographic consequences of infections. The ability of rodents to be infected with an orthohantavirus without noticeable effects does not alone implicate them as a reservoir. Virus isolation is generally deemed the gold-standard evidence to support a reservoir role, followed by positive polymerase chain reaction (PCR) results. While orthohantavirus isolation from rodent hosts is rare—even for well-established virus-host relationships (e.g., SNV and *Peromyscus maniculatus* [22])—recent advances in establishing rodent cell cultures, such as those of the BCCV host *Sigmodon hispidus*, may aid future isolations [23]. In contrast, positive RT-PCR results for a particular virus in multiple rodent species are common (Table A1).

Orthohantavirus infections are generally considered single-host-single-virus systems [24,25,26], and viruses tend to co-diverge with their hosts [27]. The term “primary host” is sometimes used for the most common reservoir host [25,28], but this wording retracts from the idea that orthohantaviruses could persist in multiple hosts with the same propensity. Evidence increasingly suggests that some American orthohantaviruses do not follow the single-host-single-virus paradigm as strictly as their Old World counterparts (Table A1). For example, the reservoir for Lechiguanas virus (LECHV) is considered to be *Oligoryzomys flavescens*, but results from a recent study found LECHV-positive reverse transcriptase PCR (RT-PCR) samples from *Oligoryzomys nigripes* in Argentina, while all *O. flavescens* samples were seronegative [29]. Similarly, a study in Texas found SNV-positive RT-PCR samples from five seropositive *Peromyscus attwateri,* four *P. leucopus*, one *P. laceiarus,* and one *Reithrodontomys fulvescens*, but all of the sampled *P. maniculatus*, the reservoir of SNV, were seronegative except a single RT-PCR negative individual [17]. It is unknown whether such instances are caused by frequent spillover events or the persistence of the virus within or among multiple species.

Multiple-host systems are also more common than generally acknowledged when considering virus genotypes that are not classified as separate viruses by the ICTV. In such cases, the reservoirs for a virus strain would be the combination of reservoirs for all genotypes. For example, Limestone Canyon virus (LSCV) is a genotype of SNV that is associated with *Peromyscus boylii* and other *Peromyscus* species [30,31] instead of *P. maniculatus,* the reservoir of SNV; Isla Vista virus (ISLAV) is a genotype of PHV that is associated with *Microtus californicus* [32] instead of *M. pennsylvanicus,* the reservoir of PHV; and Rio Mearim virus (RIMEV) and Anajatuba virus (ANAJV) are genotypes of Rio Mamoré virus (RIOMV) that are associated with *Holochilus sciureus* and *Oligoryzomys fornesi*, respectively [33], instead of *O. microtis,* the reservoir of RIOMV (Table A1). In some circumstances, distinct orthohantavirus genotypes are also host subspecies-dependent. For instance, *Oryzomys couesi* is suggested to be the reservoir for Catacamas virus (CATV) and Playa de Oro virus (OROV), a genotype associated with a clade composed of CATV, BCCV, and Bayou virus (BAYV), but OROV and CATV are associated with different subspecies of *O. couesi* [34,35]. Choclo virus (CHOV) and Maporal virus (MAPV) are both associated with *Oligoryzomys fulvescens*, although a distinction in the mitochondrial cytochrome-*b* gene suggests that these viruses are host subspecies-specific, infecting *O. f. costaricensis* and *O. f. delicatus*, respectively [36].

**Figure 1:**
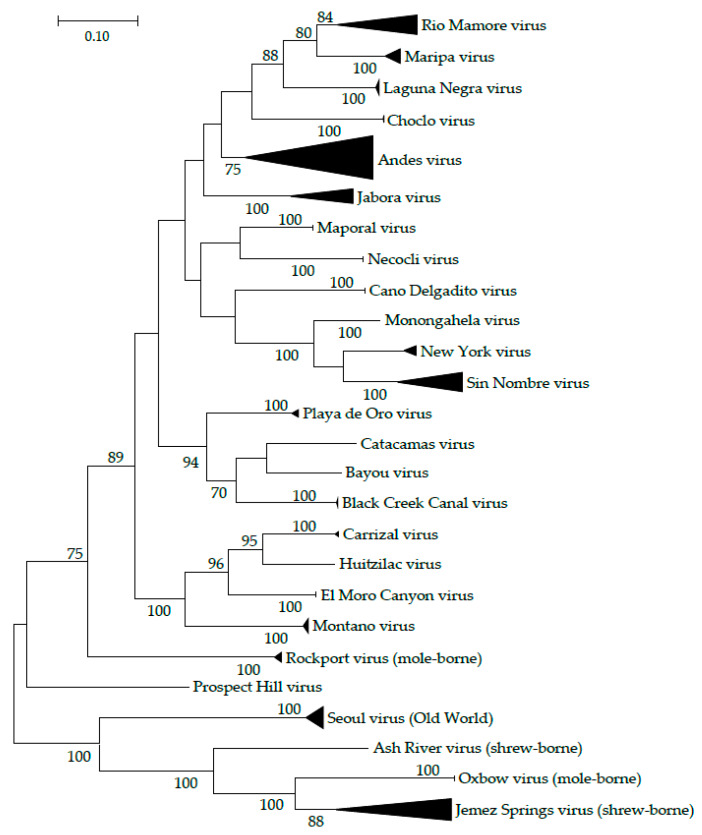
Phylogenetic tree demonstrating relatedness among American rodent-borne orthohantaviruses. The evolutionary history was inferred using the Maximum Likelihood method implemented in MEGA7. The percentage of trees in which the associated taxa clustered together is shown next to the branches; values over 70% are shown. The tree is drawn to scale, with branch lengths measured in the number of substitutions per site. Triangular branches represent multiple closely-related sequences. The analysis involved 111 orthohantavirus S segment nucleotide sequences retrieved from GenBank.

Another technical issue to consider is the concept of host-switching events among orthohantaviruses. Evidence of historical host switching has resulted in hantavirus lineages among disparate mammal taxa [37,38,39]. More recent host-switching is supported by several mismatches in the cophylogeny of orthohantaviruses and rodent hosts. For example, Monongahela virus (MGLV) is an orthohantavirus genotype primarily carried by *P. maniculatus nubiterrae*, a subspecies of the mouse associated with SNV, despite MGLV often showing a closer phylogenetic relationship to New York virus (NYV), which is associated with *P. leucopus* [40,41,42] (although the true relationship is still unknown (Figure 1), and MGLV has also been reported in *P. leucopus* [43]). Additionally, OROV and CATV are often found in the same species, *O. couesi*, despite OROV being more closely-related to BCCV, which is associated with *S. hispidus*. Minimal range overlap between *O. couesi* and *S. hispidus* and minimal genome sequencing prevent conclusion of a host-switch event among these viruses. 

Additional host-switching events have been proposed as the reason for the multitude of reported hosts in American orthohantaviruses (Table A1), such as from *Oligoryzomys flavescens* to *O. nigripes* for LECHV [29]. However, while reports of orthohantaviruses infecting multiple species supports multiple hosts for many orthohantaviruses and, therefore, a plethora of host switching events, there is a shortage of research examining the competence of many putative hosts and therefore the classification of true reservoirs. Information regarding the relative transmissibility of virus from each host to humans and other wildlife is also lacking, with the exception of several case studies involving focused trapping around areas of assumed exposure (e.g., [42,44]). Additionally, no orthohantavirus has been isolated from more than one rodent species (Table A1), although few studies have reported such attempts. Further research is, therefore, necessary to determine if frequent documentation of American orthohantaviruses in multiple species represents host switches or spillover.

## 3. Orthohantavirus Communities

In addition to the potential for multiple hosts, the number of sympatric viruses must also be considered. Propensity for coexistence of different orthohantaviruses within a rodent community appears to vary spatially and temporally. In one Texas study, viruses, and even virus genomes, appear to segregate at the county level [17]. Similar results were found in Mexico, with most states containing only one orthohantavirus [30], although another study examining a smaller portion of the same Mexican region found viruses to commonly coexist [45]. Sympatric RIOMV genotypes, ANAJV and RIMEV, were also found in the same area but in distinct host species [33]. In California and Nevada, ELMCV, PHV, and SNV were also found in the same area, indicating that viruses hosted by diverse rodents can exist in sympatry [46]. Thus, multiple orthohantaviruses may exist together in rodent communities, but separation based on habitat type and species distributions likely play a role in structuring their presence. 

In the absence of data on orthohantavirus presence in a particular area, host distributions may be useful as proxies, as rodent ranges and habitat types are often well-documented [25,47,48]. Several orthohantaviruses have been found throughout large extents of their host range, including BCCV [17,49], BAYV [50,51], and others, indicating that orthohantaviruses have the potential to be present throughout the entire range of host species. However, the use of virus genotypes causes confusion when determining the range of orthohantaviruses. For example, BCCV is used in Florida, United States [49] while MULV is used in Texas [17]. Similarly, CHOV is used in Panama [52] while its genotype Jabora virus (JABV) is used in Brazil [53]. Until such genotypes are considered distinct viruses by taxonomists (i.e., ICTV), acknowledgement of these relationships may be helpful in minimizing confusion and aiding understanding of orthohantavirus distributions.

Without analyzing positive samples throughout species ranges for new viruses, incorrect assumptions may also be made regarding orthohantavirus distributions. For example, RIOMV infects *Oligoryzomys microtis* throughout most of its range in South America [47], so HCPS cases in French Guiana were thought to be RIOMV [35]. However, virus sequencing from an HCPS case in French Guiana found that Maripa virus (MARV), a then-new virus closely-related to RIOMV found in *O. fulvescens* and *Zygodontomys brevicauda*, was the responsible agent [54,55,56]. Difficulty in estimating virus range via host range also increases when one species can host several viruses. Both Necocli virus (NECV) [57] and MARV [55] have been found in *Z. brevicauda* via positive RT-PCR, but the range of each particular virus is unknown. A similar situation was found for *O. longicaudatus,* the most common host of ANDV and also the host of Oran virus (ORNV), although the increased attention given to ANDV revealed which populations of *O. longicaudatus* host which virus [48]. Therefore, host distribution can be useful in estimating virus distribution, but caution should be applied. 

**Figure 2:**
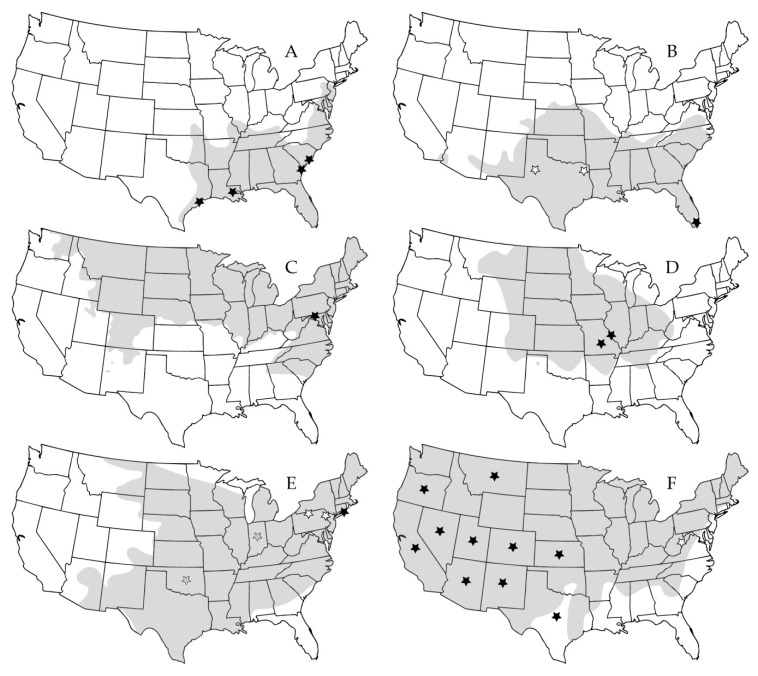
Distribution of rodents associated with orthohantaviruses that inhabit grasslands in the United States and locations where orthohantavirus-positive individuals have been found. (**A**) *Oryzomys palustris*; black stars indicate Bayou virus. (**B**) *Sigmodon hispidus*; black star indicate Black Creek Canal virus, white stars indicate Muleshoe virus. (**C**) *Microtus pennsylvanicus*; black star indicates Prospect Hill virus. (**D**) *Microtus ochrogaster*; black stars indicate Bloodland Lake virus. (**E**) *Peromyscus leucopus*; black star indicates New York virus, white stars indicate Monongahela virus, striped stars indicate Blue River virus at the state level. (**F**) *Peromyscus maniculatus*; black stars indicate Sin Nombre virus at the state level, white star indicates Monongahela virus. Distribution ranges were taken from International Union for Conservation of Nature (IUCN) Red List.

**Figure 3:**
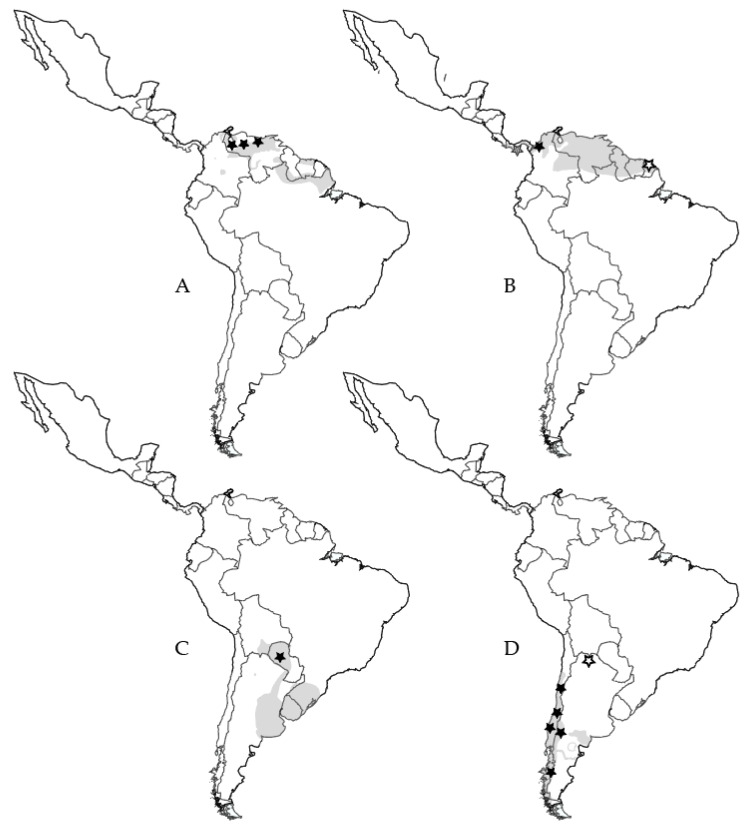
Distribution of rodents that inhabit grasslands in Central and South America and locations where orthohantavirus-positive individuals have been found. (**A**) *Sigmodon alstoni*; black stars indicate Caño Delgadito virus. (**B**) *Zygodontomys brevicauda*; black star indicates Necocli virus, white star indicates Maripa virus, striped star indicates Calabazo virus. (**C**) *Calomys laucha*; black star indicates Laguna Negra virus. (**D**) *Oligoryzomys longicaudatus*; black stars indicate Andes virus, white star indicates Oran virus. Distribution ranges were taken from IUCN Red List.

Hantaviruses are likely to spread to new areas and vanish from existing areas due to changes in rodent host distribution and abundance. Changes in grassland habitats caused by land-use changes and climate change [58,59,60] have been strongly associated with rodent distributional changes. For example, range expansion of a North American grassland rodent species, *Baiomys taylori*, was recently found in New Mexico, United States, likely due to an increase in grassland areas, particularly along roadsides, due to climate change and habitat disturbance [61]. Thus, the grassland rodents that host orthohantaviruses may show similar patterns in the future. Several orthohantavirus hosts occupy this habitat type in the United States alone, including *M. pennsylvanicus* (PHV), *M. ochrogaster* (Bloodland Lake virus, BLLV, genotype of PHV), *Microtus californicus* (ISLAV), *Reithrodontomys megalotis* (El Moro Canyon virus, ELMCV), and *Sigmodon hispidus* (MULV and BCCV), and to a lesser extent *Oryzomys palustris* (BAYV) and *Peromyscus* spp. (NYV, SNV) (Figure 2). Many rodents known to host orthohantaviruses also inhabit other grasslands throughout the Americas (Figure 3). Similar patterns of habitat changes from land use change and climate change can be expected for habitats of other orthohantaviruses and their hosts.

## 4. Transmission among Rodents

Much of what we know about the transmission of American orthohantaviruses among conspecific rodent hosts is derived from studies of ANDV and SNV [19]. Both viruses are primarily shed in saliva, occasionally in urine, and apparently not in feces, suggesting that behaviors such as grooming and biting are the primary routes of transmission [22,62,63]. Such transmission contrasts with Old World orthohantaviruses such as Puumala virus (PUUV), which are commonly shed in feces as well [19,64]. Older males are more commonly infected with orthohantaviruses than other demographic groups [65], including SNV [20,66], Laguna Negra virus (LANV) [67], LECHV [68], and BCCV [69]. Compounding more exposure opportunities for older individuals, higher prevalence in older males is assumed to result from increased aggression and competition, primarily for access to mates [70,71]. Associations of BAYV-infected male *O. palustris* with receptive females and non-infected males with non-receptive females [51] further supports the concept of reproductive behaviors as a primary driver of orthohantavirus transmission among wild rodents. Thus, some females likely become infected via allogrooming during copulative behaviors common in rodents (e.g., [72,73]). The occasional shedding of the virus in urine may be important for transmission among conspecifics, and perhaps heterospecifics as well. Urine is used by rodents for various reproductive and territorial behaviors [74], creating ample opportunities for exposure of virus in aerosolized urine via oropharyngeal routes. However, information pertaining to virus persistence outside the host in American orthohantaviruses is limited to circumstantial evidence regarding spillover infections, and such transmission may be mitigated by uncommon virus shedding in urine.

Relatively frequent rodent spillover events (i.e., transmission from one species to another) [75] suggests that other variables, including overlap in habitat use such as shared runways, burrows, and nests, is necessary for transmission among species. During the breeding season, many rodents compete for mates, food, space, and protection of offspring, so there is little overlap in space use by conspecifics, and often congeners [76,77]. However, in the non-breeding season, these territories break down and space overlap increases [78,79]. During this time, many rodents also share burrows within [80,81] and occasionally among [82] species. During warmer months, some species may also use the burrows of other species who have since vacated [83,84]. Burrow-sharing behavior in rodents has been associated with the spread of several other diseases, including plague (*Yersinia pestis*) [85], tick-borne Relapsing Fever (*Borellia* spp.) [86], and possibly Valley Fever (*Coccidioides* spp.) [87], and the stable cool, humid microclimates of burrows [88,89] may allow orthohantaviruses to persist in the environment. This phenomenon would also help explain why multiple species can be infected by the same orthohantavirus, potential opportunities of spillover to non-muroid rodents [20], and original host-switching events to other rodents, shrews, and moles (Figure 1). Further research is necessary to determine the role of habitat overlap on conspecific orthohantavirus infection via competition, excrement exposure, and other potential sources of virus shedding and routes of transmission.

Regardless of the routes of transmission, population density appears to play a role in orthohantavirus maintenance. Experimental modeling of SNV prevalence in *Peromyscus maniculatus* populations and HCPS cases indicates that climate-mediated fluctuations in host abundance are linked to orthohantavirus outbreaks [66,90]. High seroprevalence in *P. maniculatus* is found after a time lag following high rainfall events, particularly those associated with the El Niño-Southern Oscillation (ENSO) [90,91]. Although this phenomenon has been relatively well-studied in SNV, data demonstrating similar patterns among other American orthohantaviruses is lacking. However, such lag times in other systems may explain why less-abundant species in a rodent community may occasionally be the primary carriers of orthohantavirus [52,92], as population sizes could have been larger in a recent season.

Theoretical models indicate that orthohantavirus transmission among rodents also has aspects of frequency-dependent transmission. Infection prevalence is greatly influenced by contact rates [93], which increase as population density increases. However, increases in prevalence are greater in males than in females [94], likely due to increased competitive encounters among males, but not females, at higher densities. High seroprevalence among overwintering animals [21,66] are assumed to be caused by persistently infected animals infecting susceptible individuals. Population sizes generally crash during this time period [20] (although *P. maniculatus* populations remained stable prior to the HCPS outbreak of 1998–2000 [91], likely due to a strong ENSO event), suggesting that winter infections may be caused by frequent interactions despite low host density. It is unclear how the stable winters of tropical regions impact orthohantavirus transmission systems in northern South America and Central America. Further attempts to imitate such systems in a controlled environment are necessary to better understand how orthohantaviruses persist and proliferate through rodent populations.

## 5. Risk of Spillover to Humans

Most American orthohantaviruses have been associated with at least one human case of HCPS (12/20), and approximately half (9/20) were discovered following an HCPS case (Table 1). Practically all HCPS cases are thought to be caused by spillover events from rodents to humans [19,24]. The exception comes from ANDV in Argentina and Chile where some evidence supports transmission from infected patients to family members and medical workers [95,96,97]. However, these instances are limited to outbreaks in small, rural communities, and regional medical staff that cared for HCPS patients had similar seroprevalence to the general population [98,99].

Several orthohantaviruses were originally discovered through broad surveillance of rodent tissues but were later implicated with human disease. For example, RIOMV was originally discovered while studying the host associations of *Andes orthohantavirus* strains in 1997 [100], and was connected to HCPS eight years later [33]. Other HCPS cases were attributed to the incorrect orthohantavirus until the actual virus was described, such as MARV cases originally diagnosed as RIOMV, as mentioned previously [54]. Similarly, due to regional variation in virus prevalence, ELMCV was suggested to be the etiological agent of several HCPS cases ascribed to SNV, but the virus in these cases was never tested [30]. Without verification via sequencing of HCPS cases, ELMCV is considered to not cause disease in humans. Thus, certain orthohantaviruses may be infectious to humans but incorrectly dismissed due to a lack of sequencing. Conversely, additional orthohantaviruses or viral genotypes that are pathogenic to humans may exist that have not yet been linked to any hosts, such as Tunari virus (TUNV), which was discovered following an HCPS case but the reservoir is still unknown [15].

While understanding host ecology may help explain the maintenance of orthohantaviruses in wild rodent populations, it can also inform spillover threats to humans. SNV and ANDV are both found most commonly in generalist rodent species that can be locally abundant. These host characteristics allow viruses to be present in most habitats throughout a large geographical range, increasing the likelihood of encounters between infected rodents and susceptible humans. However, due to the large number of described orthohantaviruses and their hosts, most regions and habitats have the capacity to contain multiple viruses of human health concern. On the other hand, some species and their viruses are common in a variety of habitat types. For example, in West Virginia, United States, where *Peromyscus* are the dominant muroid rodents, HCPS cases were attributed to exposure of airborne particulates of *P. maniculatus* secretions within cabins [40,42]. Such cases indicate an infection risk in seasonally-used buildings in rural areas in the northeast, similar to initial assessments in the southwestern United States [101]. Therefore, these generalist species appear to be capable of transmitting virus to humans regardless of habitat.

Urban areas may pose a risk for human exposure to orthohantaviruses and their hosts as well. For example, in addition to their abundance in forested habitats, *Peromyscus* mice are common in green urban spaces, such as the park system in New York City [102,103], and NYV was discovered on Shelter Island near New York City [18]. Notably, homeless residents may be at increased risk, as sleeping near rodent activity was associated with European orthohantavirus infections [104], although empirical evidence is lacking for American viruses. Due to limited migration of wild rodents throughout urban areas [102,103,105], green spaces may also be protected from orthohantavirus invasion. Orthohantaviruses carried by invasive rodents, such as Seoul virus (SEOV) in *Rattus norvegicus*, may pose a risk as well. SEOV has been documented in the United States and Canada due to the pet trade [106], while wild rats can also carry this virus. One study found a seroprevalence for orthohantavirus in *R. norvegicus* of 48.2% overall and 20–29.7% in green spaces in Baltimore, Maryland [107]. Although broader documentation of orthohantaviruses in urban areas is lacking, these findings as well as observations of a range of other disease-causing pathogens in urban rodents (e.g., [108,109]) suggest that this may be an area of major human health concern. While current threats would likely be documented already, misdiagnoses, failure to seek medical attention, and the potential for future outbreaks warrant attention.

It appears that HCPS risk is greatest in areas where humans infiltrate rodent habitats, rather than vice versa, such as areas of landscape fragmentation and encroachment caused by urbanization and development. In Uruguay, *Oligoryzomys flavescens* infected with LECHV were more common in disturbed habitats than in undisturbed habitats [110]. Relationships between habitat encroachment and infection risk occur for other zoonotic diseases, such as Nipah virus [4] and Ebola virus [111,112], suggesting a possible pattern in orthohantaviruses other than LECHV as well. Many forms of habitat encroachment can increase risk of exposure to orthohantaviruses. Ranching and farming activity in the Midwest United States prairies, such as construction of new barns and field plowing, could expose individuals to *Sigmodon hispidus* (BCCV); construction of rice fields and other encroachments into marsh habitat in the southern United States could expose individuals to *Oryzomyz palustris* (BAYV); the creation of edge habitat via development in the Amazon Basin provides additional habitat for *Oligoryzomys microtis* (RIOMV) and increases contact with humans. All of these rodent species carry orthohantaviruses that cause disease in humans (Table 1) [100,113,114]. 

Estimating the risk of exposure to most orthohantaviruses with various human activities in South America is more difficult due to minimal information about the ecology of the rodent hosts; although some evidence indicates that habitat disturbance, particularly construction of domiciles in rural areas, appears to increase the risk of human exposure to hantaviruses there as well [115,116]. Interestingly, ORNV-positive *Oligoryzomys longicaudatus* were found in Orán, Argentina, outside of the reported distribution range of *O. longicaudatus* (Figure 3) [117], showing the ability for agricultural development to expand orthohantavirus presence.

## 6. Conclusions

Despite the discovery of at least 20 different New World orthohantaviruses carried by rodents, most orthohantavirus studies in the Americas focus on ANDV and SNV. While the majority of HCPS cases are attributed to these viruses [118,119], recent evidence suggests that such statistics may be skewed due to misdiagnosis of either the causative orthohantavirus or of the disease itself [26,30]. We show that despite having many similar characteristics, American orthohantaviruses differ from their Old World counterparts and from each other in several ways. In the absence of empirical data, we shed light on the diversity, transmission, and risk of spillover for neglected American orthohantaviruses and viral genotypes using the ecology of their hosts and information on ANDV and SNV. Additionally, comparisons were occasionally made to Old World orthohantaviruses. The ecological approach from this review may also be useful in implicating transmission and spillover risk of Old World orthohantaviruses not yet examined.

A key constraint to inferring information about each orthohantavirus system is the complexity between the taxonomy of orthohantaviruses and their hosts. Related viruses appear to interact with hosts similarly, as shown by the comparable phylogenies of orthohantaviruses and their natural rodent hosts [24], their affinity to cause disease in humans (Table 1), and frequent spillover or multiple related hosts (Table A1). However, confusion in orthohantavirus taxonomy and the number of distinct virus strains limits further conclusions. In particular, surveillance of related rodent species may produce additional genetic samples that allow clearer orthohantavirus phylogenies to be constructed. Additional information regarding mole-borne orthohantaviruses, such as Oxbow virus (OXBV) and Rockport virus (RKPV) [120,121], and shrew-borne genotypes, such as Ash River virus (ARRV), Camp Ripley virus (RPLV), and Jemez Springs virus (JMSV) [38,122], which have similar taxonomical issues due to minimal research and have some overlap in rodent phylogeny (Figure 1), may aid in understanding rodent-borne orthohantaviruses. Ultimately, broader surveillance will aid in understanding which genotypes constitute distinct viruses and which represent genetic diversity of single orthohantaviruses.

In addition to the controversy over viral taxonomy, the ability for multiple orthohantaviruses and their hosts to persist in the same environment and region [25,47] (Figure 2) further limits conclusions on orthohantavirus samples that are not sequenced, whether rodent or human. Since multiple rodent species are commonly found RT-PCR positive for particular American orthohantavirus strains (Table A1), virus–host relationships are unclear. Although orthohantaviruses are difficult to isolate, attempts to isolate these viruses from rodent samples is necessary to determine which rodents are reservoirs and which species experience frequent spillover events. These results will aid in determining whether American orthohantaviruses follow a single-host system like their Old World counterparts. 

Empirical data on the ecology of neglected American orthohantaviruses are crucial to understanding transmission and persistence of such viruses and threats to human health. Few studies have examined the impacts of New World orthohantaviruses on rodent populations, with the exceptions of variation in prevalence between sexes and age classes [67,68,69], survivorship of age classes [20], and reproduction-dependent spatial variation [50]. Additional information regarding transmission routes and environmental persistence is also necessary, as the minimal data currently available using SNV and ANDV show mixed results [22,62,63]. 

Although HCPS cases are often associated with SNV and ANDV, changes in the landscape, climate, and host switching may cause particular orthohantaviruses to increase in severity. Each orthohantavirus may have the capability to become more significant to human health in the future, and insight into each virus is necessary for adequate preparation. Various viral families have existed amongst humans with little to no impact until recent decades. Therefore, research regarding neglected American orthohantaviruses is crucial for a holistic understanding of orthohantavirus epidemiology and to enable preparation for future risks.

## Figures and Tables

**Table 1:** American rodent-borne orthohantaviruses accepted by the International Committee on Taxonomy of Viruses (ICTV). Year described refers to the year that a description of the virus was first published. Discovery source refers to whether the virus was discovered via screening of captured wild rodents (Rodent) or through diagnostic tests of a human patient with hantavirus cardiopulmonary syndrome (HCPS).

Virus Species	Virus Strain	Virus Abbreviation	No. GenBank Submissions (Nov 9 2019)	Year Described	Human Disease	Discovery Source
*Andes orthohantavirus*	Andes virus	ANDV	285	1996	Yes	HCPS
	Castelo dos Sonhos virus	CASV	11	1999	Yes	HCPS
	Lechiguanas virus	LECV/LECHV	26	1997	Yes	HCPS
	Oran virus	ORNV	11	1998	Yes	HCPS
*Bayou orthohantavirus*	Bayou virus	BAYV	13	1995	Yes	HCPS
	Catacamas virus	CATV	3	2006	No	Rodent
*Black Creek Canal orthohantavirus*	Black Creek Canal virus	BCCV	8	1995	Yes	Rodent
*Caño Delgadito orthohantavirus*	Caño Delgadito virus	CADV	17	1997	No ^1^	Rodent
*Choclo orthohantavirus*	Choclo virus	CHOV	12	2000	Yes	HCPS
*El Moro Canyon orthohantavirus*	Carrizal virus	CARV	9	2012	No	Rodent
	El Moro Canyon virus	ELMCV	35	1994	No ^1^	Rodent
	Huitzilac virus	HUIV	4	2012	No	Rodent
*Laguna Negra orthohantavirus*	Laguna Negra virus	LANV	35	1997	Yes	HCPS
	Maripa virus	MARV	16	2012	Yes	HCPS
	Rio Mamoré virus	RIOMV	15	1997	Yes	Rodent
*Maporal orthohantavirus*	Maporal virus	MAPV	10	2004	No	Rodent
*Montano orthohantavirus*	Montano virus	MTNV	60	2012	No	Rodent
*Necocli orthohantavirus*	Necocli virus	NECV	10	2011	No	Rodent
*Prospect Hill orthohantavirus*	Prospect Hill virus	PHV	24	1985	No	Rodent
*Sin Nombre orthohantavirus*	New York virus	NYV	4	1995 ^2^	Yes	HCPS
	Sin Nombre virus	SNV	228	1994	Yes	HCPS

^1^ CADV and ELMCV have not been confirmed to be linked to any HCPS cases in humans, but circumstantial evidence suggests they may have been the causative virus in misdiagnosed cases. ^2^ NYV was first described as Shelter Island-1 virus in 1994 [18].

## Appendix A

**Table A1:** Evidence supporting natural infections of orthohantaviruses in American rodents. Bolded viruses represent strains accepted as distinct by the International Committee for Taxonomy of Viruses (ICTV), and non-bolded viruses indicate genotypes not accepted as distinct viruses by ICTV. Genotype placements are based on published phylogenetic analyses. All studies first found rodents to be seropositive for orthohantavirus antibodies and then performed reverse transcriptase polymerase chain reaction (RT-PCR) prior to sequencing or virus isolation (except for PHV, where isolation was attempted without RT-PCR). Orthohantaviruses have tri-segmented genomes—S, M, and L segments. Studies including only seropositive rodents without additional diagnostic evidence of infection were not included in this table.

Virus Strain/Genotype	Rodent Host	Genome Strands Sequenced			Virus Isolation
		S	M	L	
**Andes virus**	*Oligoryzomys longicaudatus*	X	X		X
	*Oligoryzomys chacoensis*	X	X		
	*Oligoryzomys flavescens*	X	X		
	*Abrothrix longipilis*	X			
	*Loxodontomys micopus*	X			
	*Rattus rattus*	X			
Araraquara virus	*Bolomys lasiurus*	X	X		
	*Oxymycterus judex*	X	X		
	*Akodon montensis*		X		
Juquitiba virus	*Oligoryzomys fornesi*	X	X		
	*Oxymycterus nasutus*	X	X		
	*Oligoryzomys nigripes*	X	X		
Maciel virus	*Bolomys obscurus*		X		
Pergamino virus	*Akodon azarae*	X	X		
	*Oxymycterus rufus*	X			
Tunari virus	Unknown				
**Castelo dos Sonhos virus**	*Oligoryzomys uriaritensis*	X			
**Lechiguanas virus**	*Oligoryzomys flavescens*		X		
	*Oligoryzomys nigripes*	X	X		
Bermejo virus	*Oligoryzomys chacoensis*		X		
**Oran virus**	*Oligoryzomys longicaudatus*		X		
**Bayou virus**	*Oryzomys palustris*	X	X		X
**Catacamas virus**	*Oryzomys couesi*	X	X		X
Playa de Oro virus	*Oryzomys couesi*	X	X		
	*Sigmodon mascotensis*	X	X		
**Black Creek Canal virus**	*Sigmodon hispidus*	X	X	X	X
Muleshoe virus	*Sigmodon hispidus*	X	X		
**Caño Delgadito virus**	*Sigmodon alstoni*	X	X		X
**Choclo virus**	*Oligoryzomys fulvescens (costaricensis)*	X	X		
Jabora virus	*Akodon montensis*	X			
**Carrizal virus**	*Reithrodontomys sumichrasti*	X	X	X	
**El Moro Canyon virus**	*Reithrodontomys megalotis*	X	X		
	*Reithrodontomys sumichrasti*	X	X		
	*Neotoma mexicana*	X			
Rio Segundo virus	*Reithrodontomys mexicanus*	X			
**Huitzilac virus**	*Reithrodontomys megalotis*	X	X	X	
**Laguna Negra virus**	*Calomys laucha*	X	X		X
**Maripa virus**	*Oligoryzomys fulvescens*	X	X		
	*Zygodontomys brevicauda*	X	X		
**Rio Mamoré virus**	*Oligoryzomys microtis*	X	X		X
Anajatuba virus	*Oligoryzomys fornesi*	X			
Rio Mearim virus	*Holochilus sciureus*	X			
**Maporal virus**	*Oligoryzomys fulvescens (delicatus)*	X	X		X
**Montano virus**	*Peromyscus beatae*	X	X	X	
**Necocli virus**	*Zygodontomys brevicauda (cherriei*)	X	X		
Calabazo virus	*Zygodontomys brevicauda (cherriei)*	X	X		
**Prospect Hill virus**	*Microtus pennsylvanicus*				X
Isla Vista virus	*Microtus californicus*	X	X		
	*Peromyscus californicus*	X	X		
Bloodland Lake virus	*Microtus ochrogaster*	X			
**New York virus**	*Peromyscus leucopus*	X	X		X
Monongahela virus	*Peromyscus maniculatus nubiterrae*	X	X		
	*Peromyscus leucopus*	X	X		
Blue River virus	*Peromyscus leucopus*		X		
**Sin Nombre virus**	*Peromyscus maniculatus*	X	X		X
	*Peromyscus californicus*	X	X		
	*Peromyscus attwateri*	X	X		
	*Peromyscus eremicus*	X			
	*Peromyscus laceianus*	X	X		
	*Reithrodontomys fulvescens*	X	X		
Limestone Canyon virus	*Peromyscus boylii*	X	X		
	*Peromyscus hylocetes*	X	X		
	*Peromyscus leucopus*	X	X		
	*Peromyscus levipes*	X	X		
	*Peromyscus melanotis*	X	X		
	*Peromyscus ochraventer*	X	X		
	*Peromyscus spicilegus*	X	X		
